# The Telomerase Reverse Transcriptase (TERT) Gene Molecular Characterization in Sheep and the Association of Its Variation with Wool Traits

**DOI:** 10.3390/ani16132045

**Published:** 2026-07-03

**Authors:** Fangfang Zhao, Zhaohua He, Huitong Zhou, Hongxian Sun, Longxia Ma, Zhanchao Chen, Li Wei, Shaobin Li

**Affiliations:** 1 Gansu Key Laboratory of Herbivorous Animal Biotechnology, College of Animal Science and Technology, Gansu Agricultural University, Lanzhou 730070, China; zhaofangfang@gsau.edu.cn (F.Z.); sunhx@st.gsau.edu.cn (H.S.); 18893787530@163.com (L.M.); 1073324010058@st.gsau.edu.cn (Z.C.); 18419371725@163.com (L.W.); 2 Henan Provincial Engineering Research Center for Animal Germplasm Resources Exploration and Innovative Utilization, College of Biology and Food Science, Shangqiu Normal University, Shangqiu 476000, China; hezh1250148718@163.com; 3 Gene-Marker Laboratory, Faculty of Agricultural and Life Sciences, Lincoln University, Lincoln 7647, New Zealand; huitong.zhou@lincoln.ac.nz

**Keywords:** sheep, *TERT*, SNPs, wool traits, animal production

## Abstract

Telomerase reverse transcriptase (TERT) plays a regulatory role in the induction, activation, and hair growth of human and mouse hair follicles. However, the effects of mutations in this gene on sheep hair follicle development and wool traits remain unclear. In this study, the TERT gene (*TERT*) was investigated using Kompetitive Allele-Specific PCR (KASP) technology and in situ hybridization. The results revealed six single nucleotide polymorphisms (SNPs) across the four exons of *TERT*, two of which were non-synonymous. The *TERT* mRNA showed weak positive expression in the stratum corneum, inner root sheath, hair follicle medulla, and sebaceous glands; moderate positive expression in the outer root sheath; and strong positive expression in the dermal papilla. An association analysis between wool trait variation and the SNPs revealed that the two non-synonymous changes were associated with mean wool staple strength (MSS), thus *TERT* could potentially serve as a candidate gene for this trait.

## Simple Summary

Wool traits are mostly controlled by minor polygenes, and their developmental regulation mechanisms are complex. Accordingly, there is still a lot of work to be done in the development and utilization of marker genes for wool traits. In this study, we analyzed the expression of telomerase reverse transcriptase in the skin, variation in its gene (*TERT*), and the relationship between the gene and selected wool traits. The results suggest that *TERT* is widely expressed in the skin, and that variations in this gene are associated with average wool strength, although further analysis of other flocks is needed to confirm these findings.

## 1. Introduction

Telomerase reverse transcriptase (TERT) is the protein component of telomerase. It can maintain telomere function by adding repeat sequences to the ends of chromosomes and is mainly expressed in stem cells and progenitor cells [1,2]. The TERT gene (*TERT*) is located on chromosome 5 in humans and chromosome 16 in sheep. It plays an important role in maintaining telomere length, maintaining telomere function, ensuring cell viability and chromosome stability [3]. Studies have revealed that changes in methylation of the *TERT* promoter are the main mechanism for upregulating telomerase, and this plays a key role in the development of tumors and various types of human cancer [4]. It may promote the transition of hair follicles from telogen phase to anagen phase [5]. In addition, conditional activation of *TERT* in mouse skin can induce the proliferation of skin hair follicle stem cells, thereby rapidly converting hair follicles from the resting phase to the growth phase [6]. Overexpression of *TERT* can improve the viability of dermal papilla cells, which is beneficial to the improvement of its ability to induce mouse hair follicles [7]. Related studies have found that the presence of *TERT* expression in skin keratinocytes activates dormant hair follicle stem cells, thereby activating hair follicles and promoting hair growth [8,9]. The above studies suggest that *TERT* has a regulatory effect on the induction and activation of hair follicles, and thus hair growth in humans and mice. However, the impact of this gene on wool follicle development and wool traits in sheep is still unclear.

## 2. Materials and Methods

### 2.1. Ethics Statement

The sheep used in the study were from a farm in Tianzhu Zangzu Autonomous County, China. Informed consent was obtained from the sheep owner. The animal experiments were approved by the Gansu Agricultural University Ethics Committee with the approved code GSAU-LC-2020-019.

### 2.2. Sheep and Sampling

Blood and wool samples were collected from 315 1-year-old Gansu alpine fine wool ewes in Songshan Town, Tianzhu Tibetan Autonomous County, for the identification of single nucleotide polymorphisms (SNPs) and subsequent association analysis with wool traits. All the sheep had consistent nutritional levels and feeding environments. The collected blood samples were stored at −20°C for subsequent use in blood DNA extraction.

Wool samples were collected from the left scapula to determine various wool traits such as mean fiber diameter (MFD), coefficient of variation in fiber diameter (CVFD), standard deviation of fiber diameter (FDSD), mean fiber curvature (MFC), comfort factor (CF), mean fiber length (MSL), and mean fiber staple strength (MSS). This analysis was conducted by the New Zealand Wool Testing Authority (Ahuriri, Napier, New Zealand) using International Wool Textile Organization (IWTO; https://iwto.org/)-endorsed methods (https://iwto.org/resources/wool-testing-resources/; accessed on 15 September 2023).

To ascertain the location of expression of TERT in ovine skin, one sheep was randomly selected for skin tissue sampling to facilitate subsequent in situ hybridization localization analysis of target genes. Skin tissue was collected from a 5 cm^2^ area of the posterior border of its scapulae after localized injection of lidocaine hydrochloride (Suicheng Pharmaceutical Co., Ltd., Zhengzhou, China) at the site of collection for anesthesia. To prevent infections, Yunnan Baiyao (Yunnan Baiyao Group Co., Ltd., Kunming, China) and penicillin (Jiangxi Ruicheng Hongbao Veterinary Medicine Co., Ltd., Nanchang, China) were applied to the wound after the sampling.

### 2.3. Gene in Situ Hybridization Localization Analysis

After 48 h of fixation in situ hybridization fixative, sheep skin tissue was subjected to in situ hybridization experiments using the method reported by Sun et al. [10]. Using target gene probe hybrids for mRNA expression localization test, and the probe sequence (5′ → 3′) was “ACTGGATGTAGGACCTGCCCCCGAT”, and hybridization temperature was 40 °C. CaseViewer2.4 (3DHISTECH, Budapest, Hungary) image analysis software was used to observe microscopic images for analysis, then perform gene mRNA positive expression scoring on the stratum corneum, dermal papilla, sebaceous gland, inner root sheath, outer root sheath, and medullary areas within the three fields of view of the slices.

The core indicator for expression determination is the intensity of the positive staining signal, which was divided into the following categories: −: no positive expression; +: weak positive expression; ++: moderate positive expression; +++: strong positive expression. In detail, “−“: no specific staining was observed in the full-thickness skin (epidermis, dermis, appendages), which was consistent with the background. “+”: the signal is weak and needs to be magnified to 200× or 400× to be clearly distinguishable. “++”: Clearly visible under a 100× magnification, with a clear signal. “+++”: It is immediately apparent under low magnification (40–100×), showing a patchy distribution.

### 2.4. Extraction of Blood Genomic DNA

The EasyPure ^®^ Blood Genomic DNA Kit (Beijing Quanshijin Biotechnology Co., Ltd., Beijing, China) extraction kit was used to extract genomic DNA from blood. Agarose gel electrophoresis was used to detect DNA quality, and NanoDropTM Lite ultra-micro spectrophotometer (Thermo Fisher Scientific, Waltham, MA, USA) was used to detect the concentration and purity of genomic DNA. This DNA was stored at −20 °C for future use.

### 2.5. Detection of SNPs in TERT Gene

The ENSEMBL database was used to predict the nature and type of polymorphism in *TERT* and select the target fragments to be amplified. After retrieval of the entire sequence of sheep *TERT* (NC_056069.1, gene ID: 100126866) from NCBI, it was used to design specific primers using Premier 5.0 software (Table 1). The primers were synthesized by Sangon Biotech, Shanghai, China.

This study selected genomic DNA from 20 Gansu Alpine Fine wool sheep and amplified the target fragment of *TERT*. Agarose gel electrophoresis was used to detect the integrity of amplification products and conducted by Wuhan Gentides Biotechnology Co., Ltd. (Wuhan, China), then sent the amplification products to Sangon Biotechnology, Shanghai, China for first generation Sanger sequencing. After sequencing was completed, the SNP sites of the target fragment were screened by combining the sequencing peak map with the sequence information of the bases.

### 2.6. Detection of Genotype

Genotyping was conducted by the Wuhan Gentides Biotechnology Co., Ltd. using kompetitive allele-specific PCR (KASP), and according to the SNP sites detected in the step above. The collection of fluorescence data uses a microplate reader with a fluorescence resonance energy transfer (FRET) probe. A genotyping map was generated using online software SNPDecoder. In KASP genotyping experiments, individual samples failing to be successfully genotyped are mostly due to insufficient DNA concentration of the sample, inadequate purity (residual PCR inhibitors), or anomalies in single-well operations (such as missing reagents, liquid evaporation, or cross-well contamination). These factors can interfere with specific PCR amplification and fluorescent signal collection, ultimately preventing the acquisition of effective genotyping results.

### 2.7. Statistical Analysis

Microsoft Excel 2021 was used for organizing and statistical analysis of SNP genotype data and based on the method reported by Botstein et al. [11], allele frequency (AF), genotype frequency (GF), chi-square (α2), gene heterozygosity (He), homozygosity (Ho), polymorphism information content (PIC), and effective number of alleles (Ne) were calculated. Only genotypes with a GF greater than 0.03 were used for subsequent association analysis with wool production traits. Online software SHEsis (https://github.com/celaoforever/SHEsisPlus, accessed on 29 June 2026) was used to analyze the linkage disequilibrium (LD).

The general linear model (Y_j_ = μ + G_i_ + e_j_) in SPSS 26.0 was used to analyze the relationship between genotype and wool production traits. Y represents the phenotypic value of the trait; µ was the overall mean; G_i_ was the genotype effect; e_j_ was the random error. Bonferroni correction was applied to correct raw *p*-values for multiple pairwise comparisons in the general linear model. Corrected *p*-values < 0.05 were considered statistically significant. The experimental results were expressed as mean ± standard error.

## 3. Results and Analysis

### 3.1. Localization of Expression of the TERT Gene in Skin Tissue of Gansu Alpine Fine Wool Sheep

**Figure 1:**
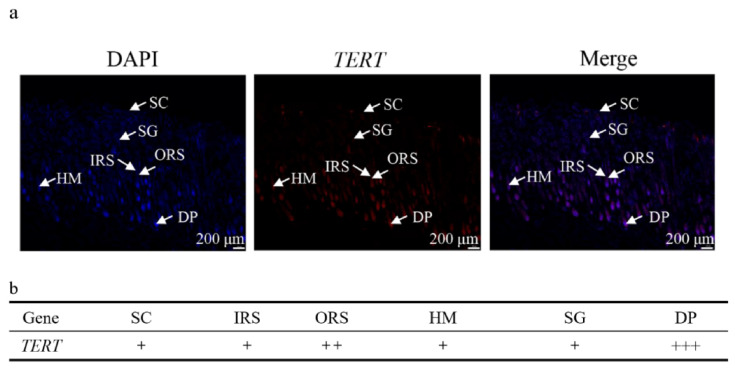
In situ hybridization staining (**a**) and positive intensity (**b**) of the *TERT* in skin tissues of Gansu alpine fine-wool sheep. Note: 1. *TERT* fluorescence staining is red, and DAPI-labeled cell nucleus fluorescence staining is blue. SC: Stratum corneum; IRS: Inner root sheath; ORS: Outer root sheath; HM: Hair medulla; SG: Sebaceous gland; DP: Dermal papilla. 2. +: Weak positive expression; ++: Moderate positive expression; +++: Strong positive expression.

The results of in situ hybridization reveal that the mRNA of the TERT was weakly positively expressed in the stratum corneum, inner root sheath, hair medulla, and sebaceous gland, moderately positively expressed in the outer root sheath, and strongly positively expressed in the dermal papilla (Figure 1).

### 3.2. PCR Amplification Product Detection

**Figure 2:**
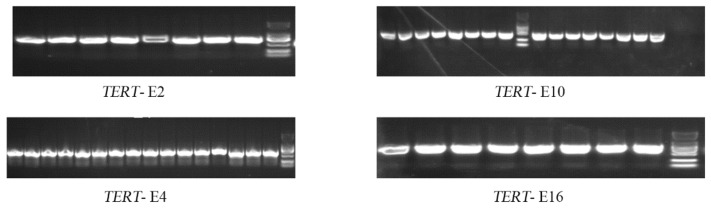
Electrophoresis of amplified fragments of *TERT* from Gansu alpine fine-wool sheep. Note: The marker with multiple bands in the same lane is a 2000 bp marker. The bands from bottom to top are 200 bp, 400 bp, increasing by 200 bp steps to reach 2000 bp.

Four pairs of primers were designed to amplify regions of *TERT*. Agarose gel electrophoresis of the PCR amplification products revealed that the band size was consistent with expectations, with clear bands and no non-specific bands (Figure 2).

### 3.3. Detection of SNP Loci in the TERT Gene of Gansu Alpine Fine Wool Sheep

**Figure 3:**
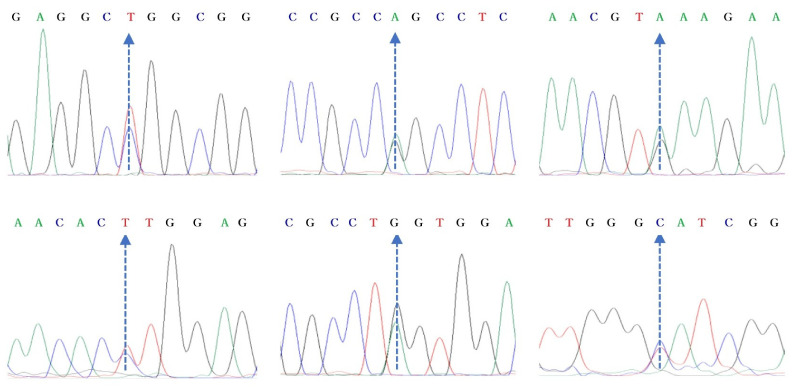
Sequencing peak maps of SNPs in the *TERT* of Gansu alpine fine-wool sheep.

The results suggest that there was a total of six SNPs in the four amplificons of *TERT* in Gansu alpine fine-wool sheep (Figure 3). The six SNPs were named SNP1 (c.1277C/T), SNP2 (c.1399G/A), SNP3 (c.1446G/A), SNP4 (c.1750C/T), SNP5 (c.2571A/G), and SNP6 (c.*197T/C), respectively. Among them, SNP1, SNP2 and SNP3 located in exon 2, SNP4 located in exon 4, SNP5 located in exon 10, and SNP6 located in 3′UTR (Table 2). 

### 3.4. Genotyping Results of SNPs Loci in TERT 

**Figure 4:**
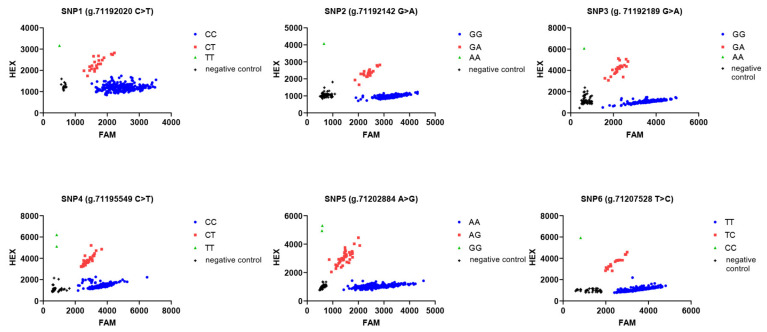
Genotyping results of *TERT* in Gansu alpine fine-wool sheep. The green and blue groups clustered near the horizontal and vertical axes represent homozygotes, respectively; the red group clustered separately between the two are heterozygotes, and the black group clustered near the intersection of the axes is a negative control.

KASP technology was used to genotype the SNPs of the *TERT* in Gansu alpine fine-wool sheep. As shown in Figure 4, all six SNPs were polymorphic and exhibited three genotypes. Due to some reactions being no-calls, some samples failed to genotype. The successful genotyping percentages for the six SNPs were: 92.70%, 95.56%, 95.56%, 95.87%, 94.29% and 95.56%, respectively.

### 3.5. TERT Genotype and Gene Frequency

The genotype and allele frequencies of *TERT* in Gansu alpine fine-wool sheep are listed in Table 3. Among them, the dominant genotypes of SNP1, SNP2, SNP3, SNP4, SNP5 and SNP6 were *CC* (91.22%), *GG* (92.03%), *GG* (91.69%), *CC* (86.09%), *AA* (85.52%), *TT* (93.69%), respectively. The dominant alleles were *C* (0.961), *G* (0.958), *G* (0.957), *C* (0.924), *A* (0.931), and *T* (0.967), respectively. The chi-square test results showed that all six SNPs of the *TERT* were in Hardy–Weinberg equilibrium (*p* > 0.05).

### 3.6. Genetic Polymorphism Analysis of TERT

The results of population genetic polymorphism showed that the homozygosity of the six SNPs in *TERT* was high (Ho > 0.5), and SNP 5 had the highest number of effective alleles (Ne = 1.163), while SNP6 had the lowest number of effective alleles (Ne = 1.069). In addition, these SNP loci were all low in polymorphism (PIC < 0.25). The results indicated that the highest heterozygous frequency occurred at the SNP5 locus (Table 4).

### 3.7. Association Analysis of TERT Gene SNPs with Wool Traits

As described in Table 5, among the 6 SNPs identified in the *TERT*, SNP1 and SNP2 were associated with MSS (*p* < 0.05). Among them, the MSS of the *CC* type in SNP1 was significantly higher than that of *CT* type individuals (raw *p* = 0.008; corrected *p* = 0.024), and the MSS of *GG* type individuals in SNP2 was significantly higher than that of *GA* type individuals (raw *p* = 0.003; corrected *p* = 0.009). Other than this, no other significant associations were noted. When the LD between SNP1 and SNP2 was analyzed, the D’ = 0.99 and r^2^ = 0.99 suggested that these two loci were in strong linkage disequilibrium.

## 4. Discussion

This study preliminarily analyzed the localization of expression of *TERT* in the skin and revealed spatial heterogeneity in the presence of mRNA in different structures of fine wool follicles from Gansu alpine fine-wool sheep. Expression was especially found in the dermal papilla area where strong positive expression was observed. This suggests that TERT may participate in the periodic regeneration process of hair follicles by regulating the telomerase activity of papilla stem cells. As the signaling center for hair follicle growth, the high expression of *TERT* in the dermal papilla may be closely related to maintaining sustained cell proliferation and delaying apoptosis, thereby affecting the growth of wool fibers. It is worth noting that moderate expression of the outer root sheath may be associated with the differentiation stage of keratinocytes in this region, while weak expression in terminally differentiated tissues such as the stratum corneum is consistent with the general inhibition of telomerase activity in differentiated cells. It should be noted that our observations were based on only one individual. In the next step, we need to expand the sample size to further verify the universality of the above results.

Among the six SNPs detected in this study, five were in exon regions, and one (SNP6) was in the 3′UTR. All conformed to Hardy–Weinberg equilibrium. The genetic equilibrium suggests that the selected population has not been significantly affected by recent selection pressure or inbreeding, and thus that the SNP variation is a stable genetic characteristic in the population. The higher frequency of SNPs revealed in the exon regions (such as exon 2 containing 3 SNPs), may suggest that these regions have been exposed to fewer functional constraints during evolution, or adaptive variations have occurred through missense mutations. However, it is necessary to clarify the specific effects of these mutations on protein structure through functional validation.

Staple strength, measured as MSS, is another crucial trait that determines wool quality. The significance of the trait is because of its association with wool processing performance. When assessing the value of fine wool, MSS is widely recognized as second only to MFD [12,13]. Wool with high MSS is commercially preferred as it is easier to spin, rarely causes machine downtime, and ultimately results in stronger and more uniform yarn [14,15]. Especially during the early stages of wool processing, wool with lower MSS produces shorter fibers, which often leads to surface fuzzing and pilling in clothing fabrics. Accordingly, sheep with *CC* at SNP1 and *GG* at SNP2 may be more popular with textile manufacturers. The limitation of this study does however lie in the lack of information about the study population, such as its parental background and sheep’s live weight, etc. If more factors affecting wool production could be incorporated into the model, the analysis results would be more credible.

Many studies have reported the close interaction between Wnt/β-catenin and hTERT. Some research reported that *TERT* had been identified as a downstream target of the Wnt/β-catenin pathway while some suggested that TERT acted as a transcriptional regulator of this pathway [16,17,18]. Given that the Wnt/β-catenin pathway is a central regulator of hair follicle morphogenesis and the hair cycle, its activation is crucial for initiating and sustaining the anagen stage. Non-synonymous mutations in this gene may alter its encoded protein, which in turn affects its function, subsequently influencing hair follicle development and wool traits. As the two SNPs associated with MSS were in the same exon, and were in strong linkage disequilibrium, it could be speculated that they create functional variations within the same haplotype block, with both influencing the expression level of the target gene. However, the regulatory mechanisms involved still require extensive follow-up research.

## 5. Conclusions

Preliminary observations suggest that the mRNA of *TERT* was widely expressed in the skin, but there were differences in expression among different sites. A total of six SNPs were detected across four regions of the gene, two of which were nonsynonymous. These two SNPs were associated with mean wool staple strength (MSS). The gene *TERT* could possibly be exploited as a candidate gene for wool MSS.

## Figures and Tables

**Table 1:** Primer information for amplifying regions of the *TERT* gene.

Gene	Position	Forward Primer (5′ →3′)	Reverse Primer (5′ → 3′)	Amplification Length (bp)
*TERT*	E2	GGAAACCATCTTTCTGGACTCGAAGC	GATGAACCACCGAAGCCACGC	717
	E4	CCACCAGCCTCCTTCAGACCC	CAGGAGACGGGGGCACAGAG	612
	E10	GAGCCGAATCCCATGGACACTG	AGGCCAGCCCTGAAACGTGAG	437
	E16	GTGATGGGTTGTCTCCCCTTGC	AGAAGGCGTTGACCGAGGGC	778

**Table 2:** SNPs related information of *TERT*.

SNPs	Position	Nucleotide Position	Variation Type	Amino Acid Change
SNP1	Exon 2	c.1277	C → T	p.P426L
SNP2	Exon 2	c.1399	G → A	p.G467S
SNP3	Exon 2	c.1446	G → A	No change
SNP4	Exon 4	c.1750	C → T	No change
SNP5	Exon 10	c.2571	A → G	No change
SNP6	3′UTR	c.*197	T → C	No change

**Table 3:** Genotype frequency and allele frequency of *TERT*.

SNPs	Genotype Frequency (*n*)			Allele Frequency		χ^2^	*p*
SNP1	*CC*	*CT*	*TT*	*C*	*T*	0.716	0.397
	0.925 (270)	0.072 (21)	0.003 (1)	0.961	0.039		
SNP2	*GG*	*GA*	*AA*	*G*	*A*	0.485	0.486
	0.920 (277)	0.076 (23)	0.004 (1)	0.958	0.042		
SNP3	*GG*	*GA*	*AA*	*G*	*A*	0.374	0.541
	0.917 (276)	0.080 (24)	0.003 (1)	0.957	0.043		
SNP4	*CC*	*CT*	*TT*	*C*	*T*	0.115	0.735
	0.861 (260)	0.132 (40)	0.007 (2)	0.924	0.076		
SNP5	*AA*	*AG*	*GG*	*A*	*G*	1.645	0.200
	0.855 (254)	0.138 (41)	0.007 (2)	0.931	0.069		
SNP6	*TT*	*TC*	*CC*	*T*	*C*	1.436	0.231
	0.937 (282)	0.060 (18)	0.003 (1)	0.967	0.033		

**Table 4:** Analysis of genetic parameters of *TERT* gene populations.

SNPs	Ho	He	PIC	Ne
SNP1	0.924	0.075	0.072	1.082
SNP2	0.920	0.080	0.076	1.086
SNP3	0.917	0.083	0.079	1.090
SNP4	0.865	0.135	0.126	1.156
SNP5	0.859	0.140	0.130	1.163
SNP6	0.936	0.064	0.062	1.069

**Table 5:** The association analysis between *TERT* SNPs and wool traits.

SNPs	Genotype	MFD (μm)	FDSD	CVFD (%)	MSL (mm)	MFC (Number/mm)	CF (%)	MSS (cN/dT)
SNP1	*CC* (270)	21.596 ± 0.161	5.624 ± 0.074	25.065 ± 0.208	80.052 ± 0.771	104.809 ± 0.838	87.280 ± 0.853	14.276 ± 0.316 ^a^
	*CT* (21)	21.362 ± 0.461	5.505 ± 0.200	25.184 ± 0.781	78.952 ± 2.454	106.168 ± 2.542	89.526 ± 2.009	12.516 ± 0.545 ^b^
SNP2	*GG* (277)	21.541 ± 0.161	5.600 ± 0.074	25.041 ± 0.204	79.796 ± 0.763	104.976 ± 0.824	87.504 ± 0.838	14.223 ± 0.311 ^a^
	*GA* (23)	21.238 ± 0.429	5.555 ± 0.196	25.510 ± 0.810	78.652 ± 2.379	106.730 ± 2.754	89.650 ± 1.911	12.367 ± 0.509 ^b^
SNP3	*GG* (276)	21.547 ± 0.161	5.600 ± 0.074	25.041 ± 0.204	79.726 ± 0.762	104.976 ± 0.824	87.504 ± 0.838	14.214 ± 0.311
	*GA* (24)	21.220 ± 0.411	5.580 ± 0.188	25.681 ± 0.789	78.166 ± 2.329	106.910 ± 2.626	89.761 ± 1.820	12.887 ± 0.712
SNP4	*CC* (260)	21.489 ± 0.164	5.609 ± 1.092	25.090 ± 0.212	80.027 ± 0.800	104.783 ± 0.858	87.586 ± 0.860	14.123 ± 0.313
	*CT* (40)	21.670 ± 0.392	5.514 ± 0.131	25.054 ± 0.568	77.350 ± 1.647	107.180 ± 2.001	88.400 ± 1.909	14.155 ± 0.823
SNP5	*AA* (254)	21.502 ± 0.166	5.614 ± 0.081	25.062 ± 0.217	80.234 ± 0.811	104.844 ± 0.975	87.464 ± 0.881	14.163 ± 0.319
	*AG* (41)	21.612 ± 0.394	5.470 ± 0.128	25.018 ± 0.540	76.951 ± 1.643	105.929 ± 1.962	88.811 ± 1.832	13.548 ± 0.757
SNP6	*TT* (282)	21.519 ± 0.154	5.607 ± 0.733	25.125 ± 0.206	79.739 ± 0.761	105.271 ± 0.815	87.815 ± 0.793	14.052 ± 0.296
	*TC* (18)	21.393 ± 0.751	5.460 ± 0.212	24.726 ± 0.836	79.055 ± 2.245	103.046 ± 3.151	86.200 ± 3.939	14.818 ± 1.502

Notes: Different letters in the columns indicate significant differences (corrected *p* < 0.05). The results were expressed as means ± standard errors (S.E.). Individuals with rare homozygous genotypes (*TT* for SNP1, *AA* for SNP2, *AA* for SNP3, *CC* for SNP 6, *n* = 1; *TT* for SNP4, *GG* for SNP5, *n* = 2) were excluded from the association analysis to ensure statistical robustness. MFD: mean fiber diameter, FDSD: fiber diameter standard deviation, CVFD: coefficient of variation in fiber diameter, MSL: mean staple length, MFC: mean fiber curvature, CF: comfort factor, MSS: mean staple strength.

## Data Availability

Data is contained within the article. The original contributions presented in this study are included in the article. Further inquiries can be directed to the corresponding author.
